# Tracking the formation and degradation of fatty-acid-accumulated mitochondria using label-free chemical imaging

**DOI:** 10.1038/s41598-021-85795-1

**Published:** 2021-03-23

**Authors:** Chi Zhang, Stephen A. Boppart

**Affiliations:** 1 Beckman Institute for Advanced Science and Technology, University of Illinois at Urbana-Champaign, Urbana, USA; 2 Department of Bioengineering, University of Illinois at Urbana-Champaign, Urbana, USA; 3 Department of Electrical and Computer Engineering, University of Illinois at Urbana-Champaign, Urbana, USA; 4 Cancer Center at Illinois, University of Illinois at Urbana-Champaign, Urbana, USA; 5 Carle Illinois College of Medicine, University of Illinois at Urbana-Champaign, Champaign, USA; 6 Present Address: Department of Chemistry, Purdue University, 560 Oval Drive, West Lafayette, IN 47907 USA

**Keywords:** Bioanalytical chemistry, Imaging studies, Imaging and sensing, Microscopy, Optical spectroscopy

## Abstract

The mitochondrion is one of the key organelles for maintaining cellular homeostasis. External environmental stimuli and internal regulatory processes may alter the metabolism and functions of mitochondria. To understand these activities of mitochondria, it is critical to probe the key metabolic molecules inside these organelles. In this study, we used label-free chemical imaging modalities including coherent anti-Stokes Raman scattering and multiphoton-excited fluorescence to investigate the mitochondrial activities in living cancer cells. We found that hypothermia exposure tends to induce fatty-acid (FA) accumulation in some mitochondria of MIAPaCa-2 cells. Autofluorescence images show that the FA-accumulated mitochondria also have abnormal metabolism of nicotinamide adenine dinucleotide hydrogen, likely induced by the dysfunction of the electron transport chain. We also found that when the cells were re-warmed to physiological temperature after a period of hypothermia, the FA-accumulated mitochondria changed their structural features. To the best of our knowledge, this is the first time that FA accumulation in mitochondria was observed in live cells. Our research also demonstrates that multimodal label-free chemical imaging is an attractive tool to discover abnormal functions of mitochondria at the single-organelle level and can be used to quantify the dynamic changes of these organelles under perturbative conditions.

## Introduction

Mitochondria are the ‘power plants’ of cells and are involved in complex metabolic activities. Proper mitochondrial function is one of the key factors in maintaining cellular metabolic homeostasis. The ability to monitor mitochondrial dynamics and functions, and to visualize and quantify their chemical composition in living cells, is therefore essential. Mitochondria typically have diameters between 0.5 to 1 µm and lengths up to several micrometers, and hence can be seen under a light microscope. They can be visualized based on the refractive index difference between the organelle and the cytoplasm and their unique shapes. They can also be highlighted by fluorescent probes using fluorescence microscopy. However, these common ways to visualize mitochondria do not provide chemical or metabolic information. Immunofluorescence techniques can reveal the presence and amount of specific proteins associated with mitochondria, but require fixation and preprocessing of cells, and therefore cannot be used to understand extended time-lapse dynamics of these organelles. Additionally, the abovementioned imaging techniques are not selective to small metabolic molecules such as lipids and nicotinamide adenine dinucleotide hydrogen (NADH) which are key molecules involved in mitochondrial metabolism.

Raman microscopy can distinguish mitochondria from other organelles based on their unique chemical composition and is especially selective to differentiate compositional changes of small metabolites^1–3^. However, spontaneous Raman spectroscopy has long spectral acquisition times and slow imaging speeds, making it impractical to capture highly dynamic mitochondrial activities. Coherent Raman scattering microscopy techniques, including coherent anti-Stokes Raman scattering (CARS) and stimulated Raman scattering (SRS), have significantly improved the signal level as well as the speed of Raman imaging^4–12^. Both CARS and SRS allow simultaneous chemical imaging and dynamic tracking of cellular events^13–17^. The majority of coherent Raman microscopy studies have been focused on exploring the metabolism of lipid droplets (LDs)^14,18–24^, while little has been directed toward understanding mitochondrial properties.

In this study, we used CARS microscopy to image living cancer cells and discovered heterogeneity in the chemical composition of the mitochondria. We found that some mitochondria tend to accumulate fatty acids (FAs) and show strong signals of lipids after hypothermia exposure, likely induced by the perturbation on FA β-oxidation. Our multimodal imaging system also revealed enrichment of NADH molecules in these ‘FA-accumulated’ mitochondria in the multiphoton excitation fluorescence (MPEF) channel, further demonstrating the dysfunction of these organelles. Using time-lapse CARS imaging, we followed individual mitochondria trafficking and found that these dysfunctional organelles changed structures after the cells were warmed back to the physiological temperature. The labeling of cells using an autophagosome marker suggested a likely involvement of autophagy in the structural change of these organelles. This research highlights label-free multimodal chemical imaging as a unique tool to visualize the changes in chemical composition and function of mitochondria at the single-organelle level. Future studies will continue to unveil the dynamic formation of stressed mitochondria under various conditions.

## Results

### Hyperspectral CARS imaging reveals FA-accumulated organelles in stressed cells

**Figure 1:**
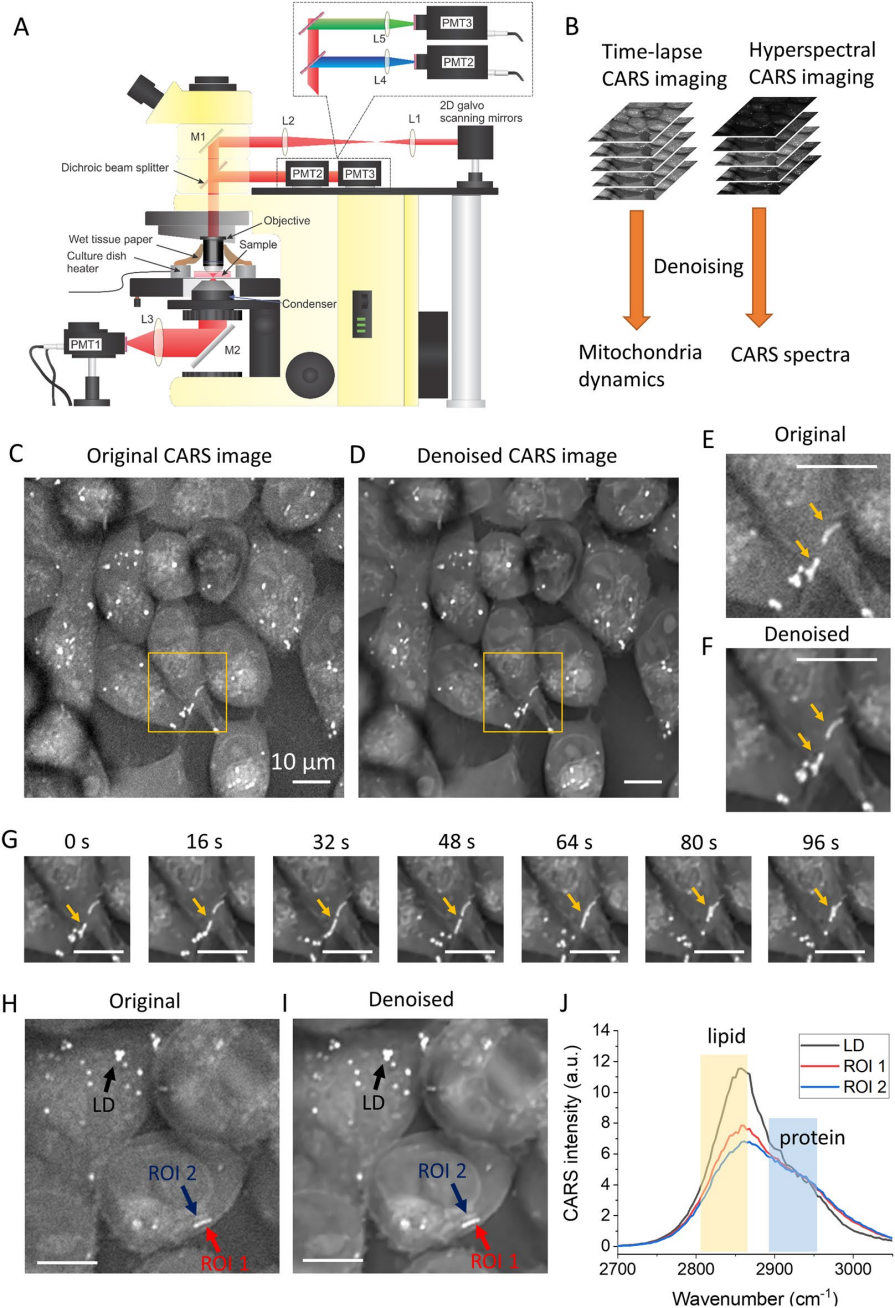
Discovery of fatty-acid-accumulated mitochondria in living cancer cells during hypothermia exposure. (**A**) Schematic of the CARS/MPEF hybrid microscope. (**B**) Illustration of denoising for hyperspectral and time-lapse image stacks. (**C**) An original CARS image at CH_2_ vibration frequency from MIAPaCa-2 cells after hypothermia exposure. (**D**) Denoised CARS image in panel (**C**). (**E**,**F**) Magnified areas indicated by the yellow boxes in panels (**C**,**D**), respectively. (**G**) Time-lapse image of the selected area in panel (**D**). Yellow arrows in panels (**E**–**G**) point to FA-accumulated mitochondria structures. (**H**,**I**) are the original and the denoised images of fixed MIAPaCa-2 cells after hypothermia exposure. Different arrows point to different targets and regions of interest (ROIs) in these images. (**J**) The original CARS spectra obtained from the hyperspectral imaging. The yellow and blue bars highlight the lipid CH_2_ and protein CH_3_ vibrational regions, respectively. *PMT* photomultiplier tube, *M* mirror, *L* lens. Scale bars represent 10 µm and apply to all images. Panel (**J**) is generated by Origin 2020.

To image organelle dynamics in real-time, we designed an integrated multimodal CARS-MPEF microscope as depicted in Fig. 1A. A dual-output laser system was used as the light source for both CARS and MPEF. The fixed-wavelength 1040 nm output was used as the Stokes beam while the wavelength-tunable output was used as the pump beam for CARS. To image lipids in the cells, we tuned the pump beam to 800 nm, corresponding to the Raman shift centered at 2884 cm^−1^. For CARS imaging, we first spatially and temporally combined the pump and Stokes beams before chirping the beams using two SF-10 glass rods (each 150 mm long). The combined and chirped beams were guided to a 2D galvo scanning system. A 40X water immersion objective lens (Olympus, LUMPlanFl, NA = 0.8) was used to focus the laser beams onto live cells cultured on glass-bottom dishes. A dish heater was installed to control the temperature at the sample. CARS signals were acquired in the forward direction using a photo-multiplier tube (PMT, Hamamatsu H7422P), while MPEF signals were collected in the epi direction using separate PMTs. For MPEF imaging of autofluorescent molecules, femtosecond laser pulses were directly used for two-photon excitation and no glass rods were implemented. Hyperspectral CARS spectra were derived using a spectral-focusing method by tuning the optical delay between the two chirped beams while collecting a single-color CARS image at each delay step^25–27^.

To better visualize mitochondrial structure and dynamics, we implemented a denoising method (CANDLE-J, ImageJ-based denoising plugin) to improve the quality of the CARS images^28^. To perform denoising, a stack of 100 time-lapse images was used (see Fig. 1B). The denoised images give better contrast for selecting the best regions of interest (ROIs) for hyperspectral CARS analysis.

MIAPaCa-2 cells were removed from an incubator and exposed to room temperature (24 °C) for about 1 h before imaging the cells with the CARS microscope. The cell dishes were sealed with Parafilm without an additional CO_2_ supply during the hypothermia exposure. We discovered the formation of rod-like structures in the cells which have strong signals in the lipid CH_2_ vibration (see Fig. 1C, inside the yellow square). A denoised image from the same region gives better contrast to highlight the lipid-accumulated rod-like structures (Fig. 1D). Figure 1E,F are magnified original and denoised images from the yellow squares in Fig. 1C,D, respectively, in which the bright rod-like organelles are visible (see arrows). The time-lapse images of this region shown in Fig. 1G reveal that these rod-like structures were transported as whole units over time, which differentiates them from aggregates of LDs, which appear as round ‘dots’ in cells and usually undergo rapid contact and dissociation over time. Supplementary Video 1 displays the original and denoised time-lapse images of Fig. 1C,D. Supplementary Video 2 displays the denoised time-lapse image of Fig. 1F. Following time-lapse imaging, we fixed the cells and acquired hyperspectral CARS images, after which we compared the CARS spectra in the C–H region for different organelles in the cells. We found that a lipid-rich rod-like organelle (see ROI 1) presents a weaker CH_2_ stretching CARS signal at 2850 cm^−1^ than the LDs, but a stronger CH_2_ signal than the structure having a very similar shape and size (see ROI 2) next to it (see Fig. 1H–J). However, if we compare the CH_3_ CARS signal at 2940 cm^−1^, the two rod-like structures have very similar intensities (Fig. 1J). Since lipids have long acyl chains with abundant CH_2_ moieties, while proteins have much fewer CH_2_ groups but a much higher portion of CH_3_ groups, our observation indicates the bright rod-like structures accumulated much more lipid molecules compared to other darker rod-like organelles, but still had less lipid composition than the LDs.

### Mitochondrion-specific fluorescent-labeling verifies mitochondria structures

**Figure 2:**
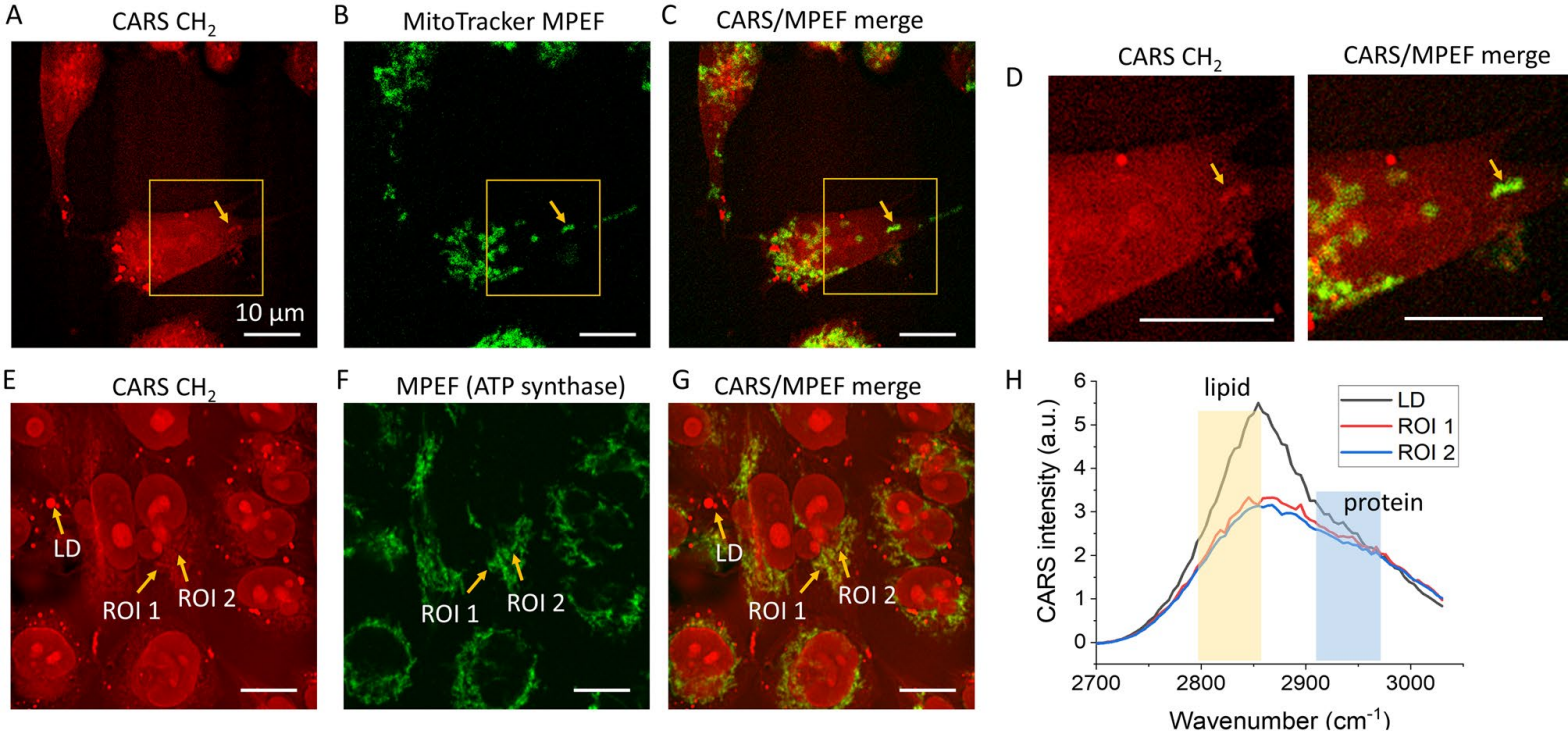
Verifying mitochondrial structures using fluorescent labeling. (**A**–**C**) CARS, MPEF, and CARS/MPEF composite images of MitoTracker labeled live MIAPaCa-2 cells, respectively. Arrows point out the target of interest. (**D**) Magnified images of areas indicated by the yellow boxes in panels (**A**,**C**). (**E**–**G**) CARS, MPEF, and CARS/MPEF composite images of immunofluorescence labeled (anti-ATPB) MIAPaCa-2 cells, respectively. Different arrows point to different targets and regions of interest (ROIs) in these images. (**H**) The original CARS spectra obtained from hyperspectral imaging. Scale bars represent 10 µm and apply to all images. Panel (**H**) is generated by Origin 2020.

To verify the structures of these organelles, we used standard fluorescence labeling. We added a mitochondria-targeted dye solution (MitoTracker Green, Cell Signaling Technology) into the culture medium of living MIAPaCa-2 cells and maintained them for 1 h at room temperature (24 °C) before imaging. As shown in Fig. 2A–C, a rod-like organelle having stronger CH_2_ signals in the CARS channel also showed a strong fluorescence signal from the MitoTracker Green in the MPEF channel 1 (571 ± 72 nm). This is distinctly different from the LDs, which are dot-like organelles that exhibit very strong signals in the CARS channel but no signals in the MPEF channel. Figure 2D shows magnified images from the highlighted square areas in the CARS and the CARS/MPEF channels.

We also used immunofluorescence to further confirm the mitochondria structures. We applied the anti-adenosine triphosphate synthase subunit beta (ATPB) antibody to target the ATP synthase in mitochondria. We then applied an Alex Fluor 488 secondary antibody (ab150113, Abcam) to visualize the mitochondria in our MPEF channel 1 (571 ± 72 nm). We compared CARS and MPEF images and selected three locations to compare the CARS spectra. The spectral signatures of LDs, mitochondria with high CH_2_ signals, and mitochondria with low CH_2_ signals (see Fig. 2H) show similar relationships as compared to our previously shown results (see Fig. 1J). Here, the CH_2_ signal differences from the mitochondria are reduced, likely due to the use of detergents which may have washed away some lipid molecules during the sample preparation.

Therefore, we hypothesize that the rod-like structures with strong CH_2_ signals are mitochondria. Mitochondria are typically protein-rich and generate weaker signals in the CH_2_ vibrations. The stronger CH_2_ CARS signals generated by some of these mitochondria indicate the accumulation of FA molecules in these organelles, which is observed for the first time. For MIAPaCa-2 cells, these structures were not observed in the physiological temperature (37 °C) but only detected after the 24 °C hypothermia exposure.

### Multiphoton autofluorescence reveals altered NADH metabolism

**Figure 3:**
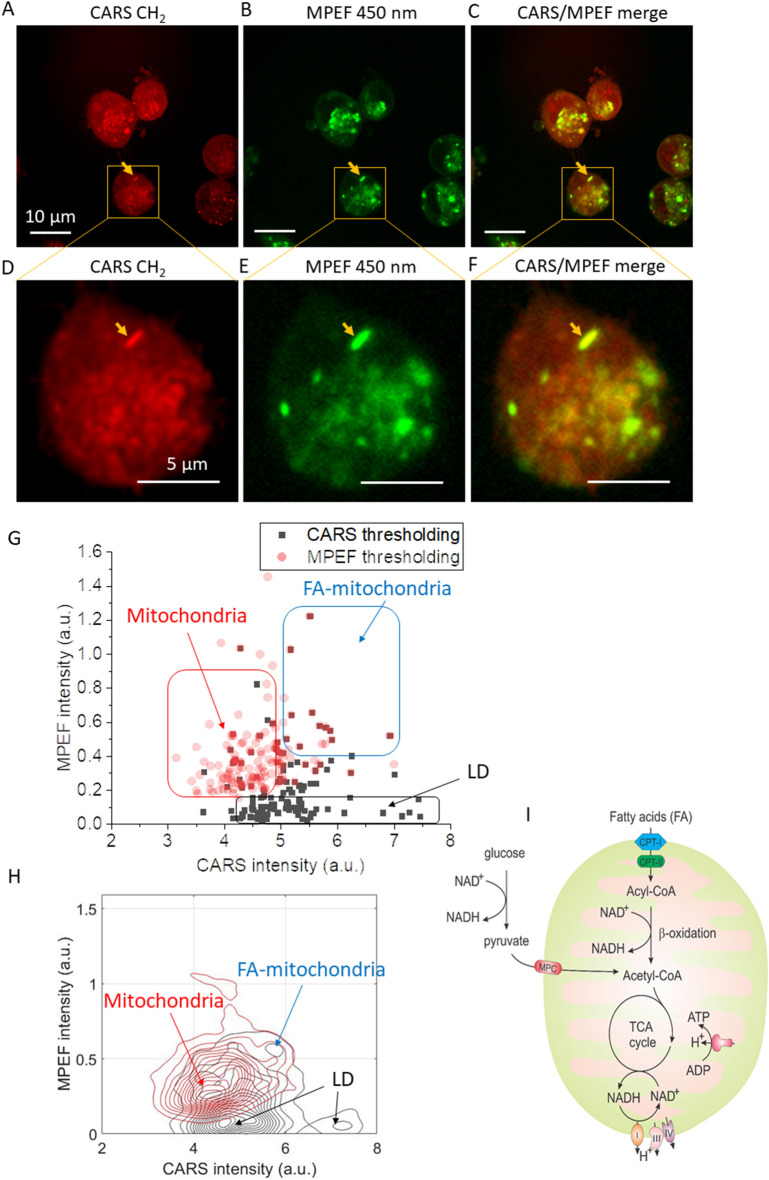
MPEF imaging revealed abnormal NADH signals in the fatty-acid-accumulated mitochondria. (**A**–**C**) CARS, MPEF, and CARS/MPEF composite images of MIAPaCa-2 cells. The MPEF detects MPEF signals from NADH centered at 450 nm. (**D**–**F**) Magnified selected areas in panels (**A**–**C**), respectively. Yellow arrows point to targets of interest. (**G**) Quantitative analysis of MPEF vs. CARS maximum intensities of detected particles by CARS and MPEF intensity thresholding. The gated areas are likely associated with LD (black), mitochondria (red), and FA-accumulated mitochondria (blue), respectively. (**H**) Contour plots of the data points in panel (**G**). Arrows point to the cluster centers corresponding to LD (black), mitochondria (red), and FA-accumulated mitochondria (blue), respectively. (**I**) Illustration of NADH metabolism related to the mitochondrion. For panels (**G**,**H**), five images were used to generate the statistical data. For the number of particles, n = 127 for the CARS thresholding, and n = 128 for the MPEF thresholding. Panel (**G**) is generated by Origin 2020, and panel (**H**) is generated by MATLAB 2020.

Mitochondria are key regulators of cellular NADH molecules. We wanted to explore if the FA-accumulated mitochondria also have abnormal NADH metabolism. NADH molecules are autofluorescent and can be excited by the 800 nm pulses through two-photon absorption and by the 1040 nm pulses through three-photon absorption^29,30^. To ensure sufficient excitation efficiency for the NADH autofluorescence, we used femtosecond laser pulses directly for both MPEF and CARS. We removed the glass rods used for hyperspectral CARS imaging in previous experiments and directly used the laser output at 800 nm and 1040 nm for CARS and MPEF. We found that the lipid-accumulated mitochondria structures show strong signals in both the transmission CARS channel and the epi-direction MPEF channel 2 (451 ± 103 nm) which detects the NADH autofluorescence emission band (Fig. 3A–C). Figure 3D–F show magnified areas in Fig. 3A–C, highlighting a target of interest (yellow arrow).

Next, we performed quantitative analyses of the CARS and MPEF intensities of different organelles. Detailed analysis procedures are illustrated in Fig. S1. We used intensity thresholding of both CARS and MPEF images to count particles in cells. For CARS intensity thresholding, we first applied a Gaussian blur filter to process the original image, and then subtract the original and the filtered image to generate a particle-enhanced image. Then, intensity thresholding was performed to isolate the particles followed by the particle analysis using ImageJ. The particle ROIs were saved and projected to the original CARS and MPEF images to obtain the maximum intensity of particles in each modality. The same processing steps were used for the intensity thresholding of MPEF images. Then, we combined the particles detected by the CARS intensity thresholding and the MPEF intensity thresholding from five images and plotted their maximum CARS vs. MPEF intensities. In Fig. 3G, the black squares are particles detected using the CARS intensity thresholding, while the red dots are particles found using the MPEF intensity thresholding. The particles in the black-gated area are likely LDs since they have relatively high CARS intensity but very low MPEF signals. The LDs with stronger CARS signals are typically larger than the LDs with weaker signals. The particles in the red-gated area, which have higher intensities in the MPEF channel but relatively low signals in the CARS channel, are likely mitochondria. The particles in the blue-gated area, with relatively strong signals in both CARS and MPEF modalities, are most likely FA-accumulated mitochondria. Intensity thresholding tends to detect only the particles having strong intensities in each modality. Therefore, the particles detected by CARS and MPEF intensity thresholding are very different. The FA-accumulated mitochondria have strong signals in both channels, and thus can be detected in both ways. In the blue gate, we found that the majority of particles are both detected by CARS and MPEF intensity thresholding (overlapped red dots and black squares). This further shows the abnormal metabolic signatures of FA-accumulated mitochondria can be identified as optical markers for the detection of these dysfunctional organelles. Figure 3H displays contour plots of the particles detected in Fig. 3G. It shows the centers of clusters corresponding to the LDs (black) and the mitochondria (red). Besides, a small and overlapped cluster also appears at the top-right of both plots, as indicated by the blue arrow. This population is contributed by the FA-accumulated mitochondria in the blue-gated area in Fig. 3G.

Figure 3J depicts FA and NADH metabolic pathways of mitochondria. It is reasonable to believe that hypothermia impacted the electron transport chain, which converts NADH to NAD^+^, and subsequently induces the accumulation of NADH in the organelles. The increased concentration of NADH possibly slows down the FA β-oxidation since NADH is the net product of this catabolic process, thus resulting in the accumulation of FA in the mitochondria.

We also compared the flavin adenine dinucleotide (FAD) level of the FA-accumulated mitochondria using the MPEF channel 1 (571 ± 72 nm), and found the accumulation of FAD molecules in these organelles (Figs. S2B and E). If we take the intensity ratio of NADH/(NADH + FAD), which is defined as the redox ratio, we find that these FA-accumulated mitochondria tend to have a higher value than other parts of the cell (Figs. S2C and F) due to the strong NADH signals, indicating an abnormal redox metabolism of these organelles.

### Degradation of FA-accumulated mitochondria at 37 °C

We found that hypothermia can induce the accumulation of FA and NADH in certain mitochondria of MIAPaCa-2 cells. These changes in organelle metabolism resulted in organelle dysfunction which might be toxic to cells. One of our recent studies showed that the LD dynamics of MIAPaCa-2 cells tend to recover quickly after a short term hypothermia exposure^31^, suggesting that the cells develop mechanisms to recover from metabolic changes induced by the hypothermia exposure. Therefore, it is reasonable to believe that cells can remove and degrade these dysfunctional mitochondria after being warmed back to 37 °C.

**Figure 4:**
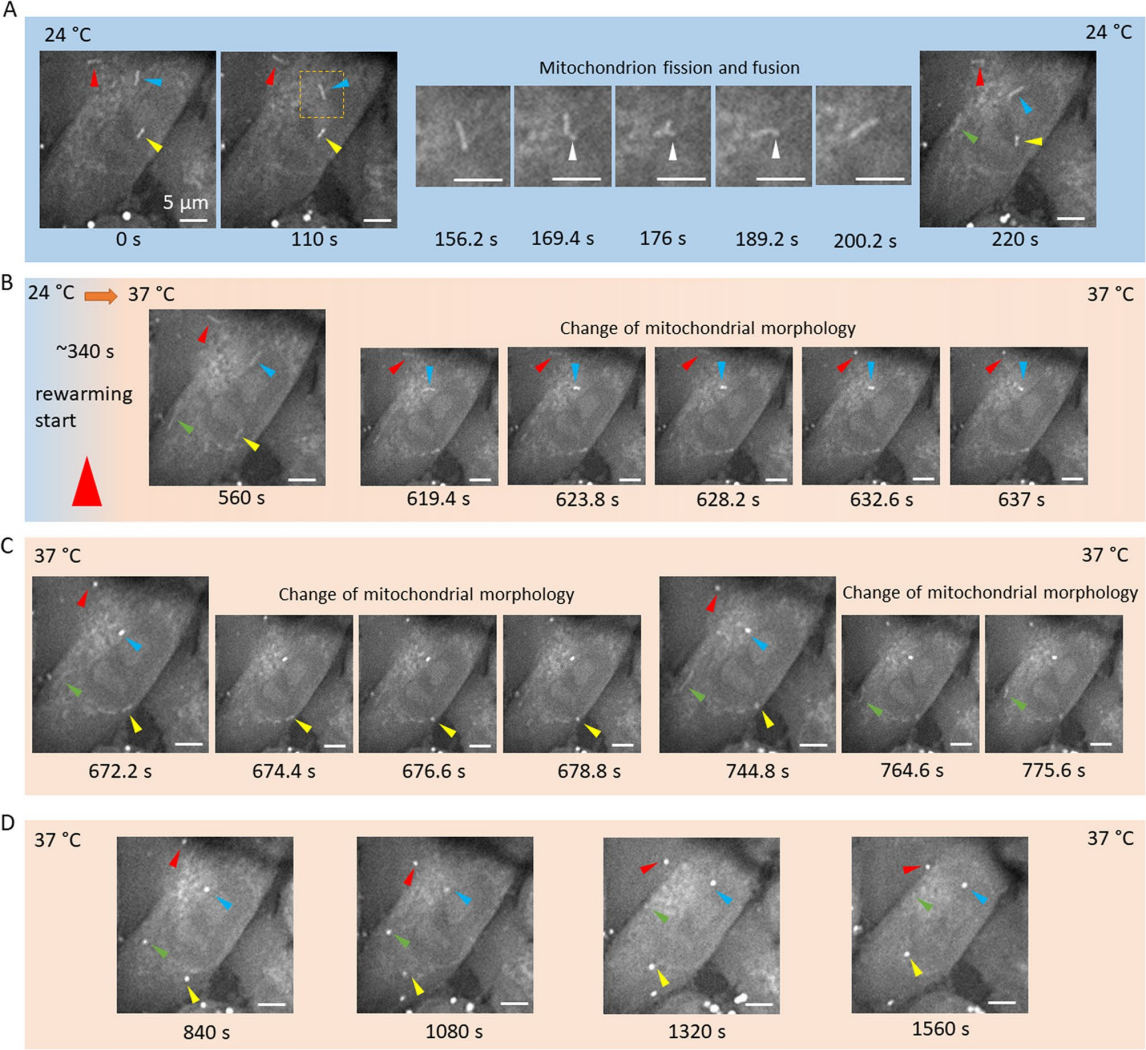
Time-lapse CARS images of a MIAPaCa-2 cell rewarming from hypothermia exposure at 24 °C to physiological temperature at 37 °C. (**A**) Imaging of the cell began in the hypothermia environment (24 °C). Rod-like FA-accumulated mitochondria were observed (colored arrows). Mitochondria fission and fusion were also observed from 156.2 s to 200.2 s (white arrows). (**B**) Rewarming started at ~ 340 s. Two FA-accumulated mitochondria were observed to change their structures. (**C**) Two additional FA-accumulated mitochondria were observed to change their structures. (**D**) All four FA-accumulated mitochondria exhibited dot-like morphology. Scale bars represent 5 µm and apply to all images in the sequence.

To observe the degradation process, we first created a hypothermia condition, allowing FA-accumulated mitochondria to form, as shown in Fig. 4A. In a MIAPaCa2 cell, we first identified 3 FA-accumulated mitochondria (see red, blue, and yellow arrows) and set this time as time 0. We found that these mitochondria underwent fission and fusion (156.2 s–200.2 s), which is a common behavior when mitochondria are under stress conditions^32–34^. Later, at 220 s, the fourth FA-accumulated mitochondrion (see green arrows) appeared. At time 340 s, we rewarmed the sample back to 37 °C. Starting from about 619.4 s, slightly less than 300 s after the rewarming started, the rod-like organelles started to form into dot-like structures (Fig. 4B–C, the blue arrow first, the red arrow second, the yellow arrow next, and the green arrow last). Eventually, all the four identified FA-rich mitochondria were converted to bright dot-like structures in the CARS image (Fig. 4D). This process might be associated with mitophagy during which mitochondria are wrapped by autophagosomes and eventually degraded by the cells^35–37^. Videos of FA-accumulated mitochondrial structural changes can be found in the Supplementary Information Video S3. These stress-induced organelles are rich in lipids and are likely recycled by the cell.

**Figure 5:**
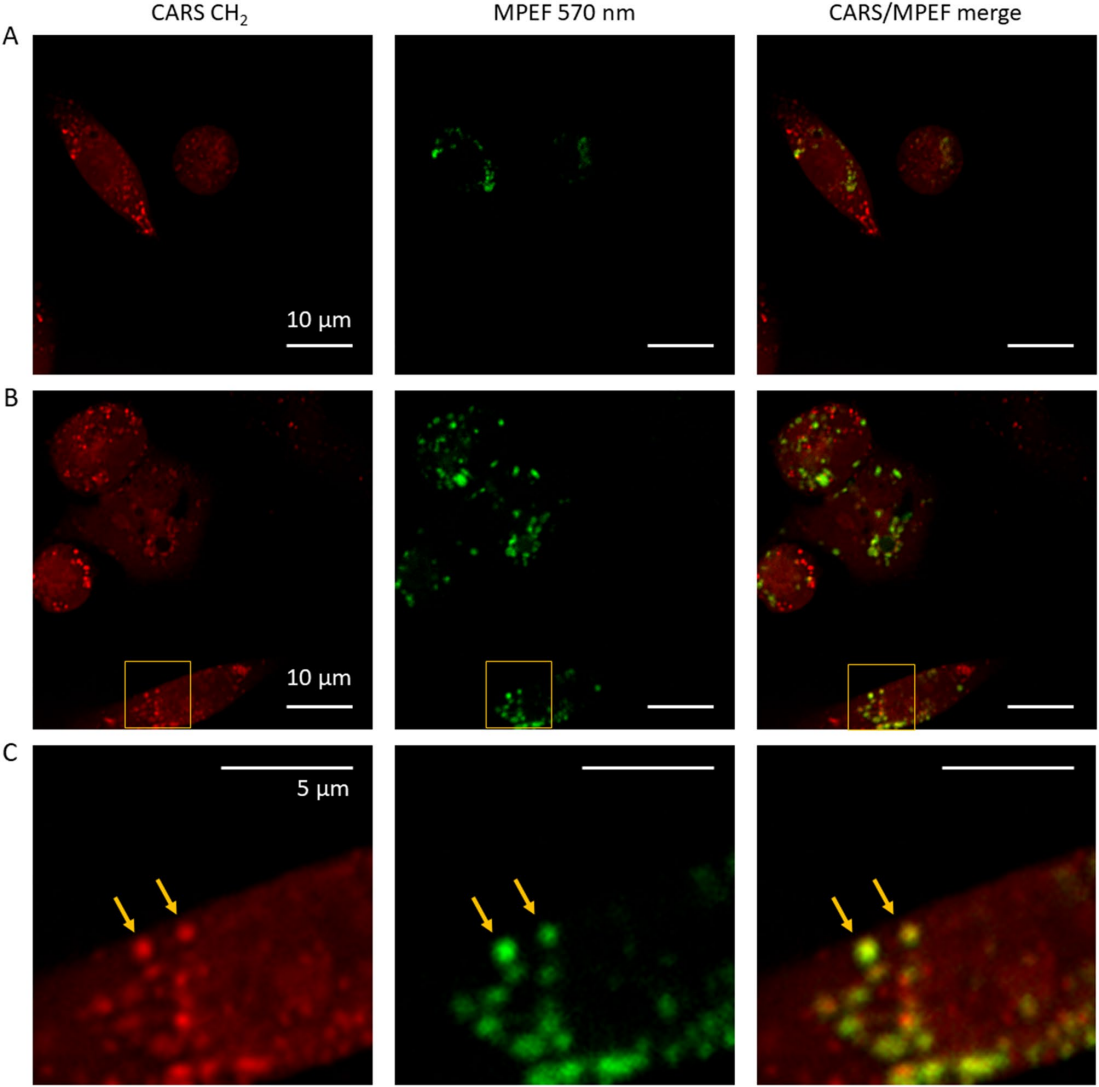
CARS and MPEF images of CYTO-ID Green labeled MIAPaCa-2 cells. (**A**) CARS (left), MPEF (middle), and CARS/MPEF (right) images of MIAPaCa-2 cells acquired at 37 °C. (**B**) CARS and MPEF images of MIAPaCa-2 cells collected at 24 °C after hypothermia exposure. (**C**) Magnified selected areas in panel (**B**). Arrows indicate molecular targets with both strong signals in CARS and MPEF.

To further verify the involvement of autophagy in the degradation of LDs, we used a CYTO-ID Green autophagy detection kit to label the autophagosomes before and after hypothermia exposure. The emission wavelength of CYTO-ID Green overlaps with FAD autofluorescence signals from cells. To separate the contribution from FAD autofluorescence, we used picosecond laser excitation. We found that compared to the CYTO-ID Green, the autofluorescence signals from cells in the MPEF channel 1 (571 ± 72 nm) could be ignored when using picosecond laser pulses for excitation. Figure 5A shows CARS and MPEF images of MIAPaCa-2 cells maintained and imaged at 37 °C. In this condition, we found no targets with overlapping strong CARS and MPEF signals. Strong CARS signals in the C–H region are mostly from LDs which do not overlap with autophagosomes in cells. However, for the cells exposed to a hypothermia environment, as shown in Fig. 5B,C, we have discovered dot-like targets with strong signals in both CARS and MPEF channels. This evidence suggests that these structures (highlighted by arrows in Fig. 5C) are likely mitophagy-related organelles similar to those observed in Fig. 4. Due to the photobleaching of CYTO-ID Green, we have not successfully tracked the morphological changes of LD-accumulated mitochondria using the MPEF signals.

## Discussion

We believe this is the first time that FA-accumulation in mitochondria has been observed and dynamically tracked over time in living cells. Such an abnormal metabolic signature is likely caused by the dysfunction of the electron transport chain, which is the primary process of converting NADH to NAD^+^. This phenomenon was only observed following the hypothermia exposure, indicating that lower temperatures tend to perturb the proper function of oxidative phosphorylation. It was reported that ADP phosphorylation is much more efficient at physiological temperature^38^. Studies also found that at lower temperatures, the maximum respiratory rates for the membrane-bound complexes were significantly reduced compared to those at the physiological temperature^39^. The increased concentration of NADH and FAD might slow down the β-oxidation process, causing the accumulation of FAs in these mitochondria^40,41^. Hypothermia might also directly affect the β-oxidation process by reducing the activity of the related enzymes^42^. Future research focusing on the inhibition and genetic knockdown of β-oxidation-related enzymes would further clarify the causes of FA-accumulation in mitochondria under hypothermia conditions.

To form FA-accumulated mitochondria, we exposed the MIAPaCa-2 cells to room temperature for 30–60 min. Based on our empirical observations, FA accumulation in dysfunctional mitochondria tends to form within an hour. Regarding the degradation, we found the change of FA-rich mitochondria started approximately 250–500 s after rewarming began. If taking the temperature rise time into account, we believe that the mitochondria structural changes start faster than this period. Our label-free chemical imaging platform offers a way to estimate the triggering time of the structural changes of dysfunctional mitochondria. During imaging, due to the use of a water dipping objective lens, we did not have a sealed chamber to maintain the CO_2_ concentration at the sample. A microscope enclosure that can better control the humidity and CO_2_ will improve future studies.

The FA-accumulation does not happen in all but in only a few mitochondrial structures in MIAPaCa-2 cells during hypothermia exposure. This limited perturbation effect likely assures the survival of cells in the hypothermia condition, and the recovery of cells after rewarming back to physiological temperature. Our results also indicate that not all the LD-like structures in the cells after environmental stress are LDs. Some of the bright dot-like structures in CARS images showing up in the CH_2_ vibrational channel (Fig. 4D) might originate from the dysfunctional mitochondria. These structures are likely to have different spontaneous Raman and CARS spectra compared to LDs. In the future, we will design experiments to identify and separate these mitophagosome structures using spectroscopic Raman techniques.

While observing FA accumulation in mitochondria, we have limited information to understand what specific types of FA are accumulated. Spontaneous Raman spectroscopy, which provides more spectral information compared to CARS, might give more spectroscopic data to probe the lipid types related to this process. To pinpoint a specific mitochondrion and collect its spontaneous Raman spectrum, we need a multimodal system with both spontaneous Raman and coherent Raman modalities, where the coherent Raman can be used to find the organelle of interest and the spontaneous Raman can be used to provide a broader-range high-resolution Raman spectrum. Such an integrated system has been reported by other groups and will be designed and built for our future studies^43^.

## Methods

### Coherent anti-Stokes Raman scattering and multiphoton excitation fluorescence microscopy

The multimodal nonlinear optical microscope integrating CARS and MPEF imaging modalities was built based on a commercial microscope frame (BX51, Olympus). A dual-output femtosecond laser system (Chameleon Discovery, Coherent Inc.) was used as the light source for signal excitation. The 1040 nm fixed wavelength output was used as the Stokes beam for CARS, while the other wavelength-tunable beam was tuned to 800 nm and used as the pump beam for CARS to excite the Raman transition at 2884 cm^−1^. MPEF excitation of NADH was primarily from two-photon absorption of the 800 nm beam, while the MPEF excitation of FAD was contributed by the nondegenerate two-photon process from the simultaneous absorption of both the 800 nm and the 1040 nm beams. The two beams were combined spatially by a dichroic beam splitter (Di02-980, Semrock) and temporally by a motorized translational stage (X-LSM050A-KX13A, Zabor Technologies Inc.). We chirped the combined pump and Stokes beams to ~ 1 ps and ~ 2 ps, respectively, by letting the combined beams pass through two SF-10 glass rods (each 150 mm long, Lattice Electro-Optics). To perform hyperspectral imaging, the motorized delay stage was swept through a 1 mm range with 10 µm per step while a single-color CARS image was acquired at each step.

A 2D galvo scanning system (8351 K, Cambridge Technology) was used to raster scan the laser beams at the sample. The water immersion objective lens used in the experiment (LUMPLFLN 40X, NA = 0.8, Olympus) was directly dipped into the culture media in cell culture dishes (µ-Dish 35 mm, ibidi). A stage-top dish heater (DH-35iL, culture dish incubator, Warner Instruments) was installed to control the temperature at the sample. To maintain the temperature and avoid media evaporation, wet tissue paper was used to cover the open areas of the dish when the heating began. Three PMTs (H7422P, Hamamatsu) were used to acquire CARS and MPEF signals. CARS signals were acquired in the forward direction, while MPEF signals were collected in the epi direction. The two MPEF channels including channel 1 (571 ± 72 nm) and channel 2 (451 ± 103 nm) were installed to simultaneously collect MPEF signals at different wavelength ranges. A black curtain was used to cover the microscope and the detectors to reduce ambient light from leaking into the PMTs. The room lights were turned off during imaging. CARS or MPEF signals detected by the PMTs were pre-amplified by a current–voltage converter and pre-amplifier (PMT-4V3, Advanced Research Instruments Corp.). Then, the amplified signals were acquired by the data acquisition system (BNC-2110 and PCIe-6351, National Instruments). The laser scanning and image acquisition were controlled by lab-written software based on LabVIEW 2019.

### Image data analysis

Single-frame or time-lapse images were acquired at a speed of 10 µs per pixel, and each image frame contained 400 by 400 pixels. Typically, the acquisition time for each CARS or MPEF image was 2.2 s. The images were saved in the .txt format. Each time-lapse sequence contained 100 2D frames. Image processing such as pseudo color conversion, channel merging, intensity value measurement, and video conversion was performed by ImageJ functions. CARS spectra data from different locations were measured by ImageJ and plotted using Origin 2020. Image size calibration was performed using 1 µm polystyrene fluorescent beads and a resolution target. Intensity thresholding was performed for both CARS and MPEF images. A Gaussian blur filter (r = 2) was first applied to the original image 5 times. Then the original image was subtracted by the filtered image to enhance the particle contrasts. Next, intensity thresholding was performed using ImageJ followed by ImageJ particle analysis. The ROIs were saved and projected to the original CARS or MPEF images to quantify the maximum intensities of each modality. The scatter plot of detected particles was created by Origin 2020. The contour plots of data points were created by MATLAB 2020.

### CANDLE-J denoising

CANDLE-J denoising was performed by ImageJ. The original image stack (100 frames) was first saved in .tiff format and then imported to ImageJ by selecting the CANDLE-J plugin. The smoothing parameters, patch radius, and search volume radius used in the processing were 0.1, 2.0, and 3.0, respectively. Denoised images were saved as .png or .avi for display.

### Cell culture

MIAPaCa-2 cells were purchased from ATCC and were cultured in Gibco Dulbecco's Modified Eagle Medium (DMEM) (4500 mg/L glucose, L-glutamine, and sodium bicarbonate) with 10% Fetal Bovine Serum and 1% Penicillin–Streptomycin (10,000 U/mL). The cells were normally cultured in a CO_2_ incubator with 5% CO_2_ concentration at 37 °C. For the hypothermia exposure, the cell dishes were sealed with Parafilm and maintained at 24 °C for ~ 1 h before imaging, or kept on the stage top incubator without heating. The cells were typically seeded to a 15 ~ 20% confluency and allow for at least 24 h to attach and grow. For the time-lapse study of mitochondria morphological change, the cells were first exposed at 24 °C to allow the formation of FA-accumulated mitochondria. Then, the cells were warmed to 37 °C by the stage-top incubator without a CO_2_ supply.

### Fluorescent labeling

MitoTracker Green was purchased from Cell Signaling Technology and used according to manufacturer instructions. A stock solution of the MitoTracker at 1 mM was directly added to the culture media to reach a final concentration of 400 nM and incubated for 1 h at 24 °C before imaging. The suggested MitoTracker concentration for incubation at 37 °C was 100–400 nM. Since we were performing incubation at 24 °C, the highest suggested concentration was used for 1 h incubation.

Anti-ATPB antibody [3D5] (ab14730, mouse monoclonal, Abcam) was used to label the mitochondria ATP synthase. Goat anti-mouse IgG Alexa Fluor 488 (ab150113, Abcam) was used as the secondary antibody to visualize the ATP synthase. The cells were first fixed for 30 min using formalin and then rinsed with phosphate-buffered saline (PBS) 3 times for 5 min each. Next, a blocking buffer (1X PBS/5% normal serum/0.3% Triton X-100) was used to treat the cells for 60 min. After blocking, the primary antibody was applied with a final concentration of 2 µg/mL. The samples were then stored overnight at 4 °C. Next, the sample was rinsed 3 times with 1X PBS for 5 min each. The secondary antibody stock solution with a concentration of 2 mg/mL was directly applied to the sample with a 1/500 dilution for 1 h to stain the ATP synthase. The sample was then rinsed before imaging.

The CYTO-ID Green was purchased from Enzo Life Sciences. The cells were originally cultured at the physiological temperature with 5% CO_2_ in the incubator. A volume of 1 µL dye was introduced to cultured cells in 1.5 mL media. For imaging at physiological temperatures, the cells were maintained in the incubator for 30 min before imaging. For imaging at hypothermia temperature, the cells were first exposed at room temperature for 20 min. Then, the CYTO-ID Green was added at 24 °C and maintained at the same temperature for 40 min before imaging at 24 °C.

## Supplementary Information

Supplementary Information 1.

Supplementary Video 1.

Supplementary Video 2.

Supplementary Video 3.

Supplementary Video 4.

Supplementary Video 5.

Supplementary Video 6.

Supplementary Video 7.

Supplementary Video 8.

Supplementary Video 9.

## References

[1] Chiu L-D, Ando M, Hamaguchi H-O (2010). Study of the ‘Raman spectroscopic signature of life’in mitochondria isolated from budding yeast. J. Raman Spectrosc..

[2] Okada M (2012). Label-free Raman observation of cytochrome c dynamics during apoptosis. Proc. Natl. Acad. Sci. U. S. A..

[3] Brazhe NA, Treiman M, Brazhe AR, Maksimov GV, Sosnovtseva OV (2012). Mapping of redox state of mitochondrial cytochromes in live cardiomyocytes using Raman microspectroscopy. PLoS ONE.

[4] Cheng J-X, Xie XS (2004). Coherent anti-Stokes Raman scattering microscopy: Instrumentation, theory, and applications. J. Phys. Chem. B.

[5] Evans CL, Xie XS (2008). Coherent anti-Stokes Raman scattering microscopy: Chemical imaging for biology and medicine. Annu. Rev. Anal. Chem..

[6] Freudiger CW (2008). Label-free biomedical imaging with high sensitivity by stimulated Raman scattering microscopy. Science.

[7] Zhang D, Wang P, Slipchenko MN, Cheng J-X (2014). Fast vibrational imaging of single cells and tissues by stimulated Raman scattering microscopy. Acc. Chem. Res..

[8] Fu D (2012). Quantitative chemical imaging with multiplex stimulated Raman scattering microscopy. J. Am. Chem. Soc..

[9] Potma EO, Evans CL, Xie XS (2006). Heterodyne coherent anti-Stokes Raman scattering (CARS) imaging. Opt. Lett..

[10] Chowdary PD (2010). High speed nonlinear interferometric vibrational analysis of lipids by spectral decomposition. Anal. Chem..

[11] Marks DL, Boppart SA (2004). Nonlinear interferometric vibrational imaging. Phys. Rev. Lett..

[12] Jones GW, Marks DL, Vinegoni C, Boppart SA (2006). High-spectral-resolution coherent anti-Stokes Raman scattering with interferometrically detected broadband chirped pulses. Opt. Lett..

[13] Shi L (2018). Optical imaging of metabolic dynamics in animals. Nat. Commun..

[14] Nan X, Cheng J-X, Xie XS (2003). Vibrational imaging of lipid droplets in live fibroblast cells with coherent anti-Stokes Raman scattering microscopy. J. Lipid Res..

[15] Zhang C, Li J, Lan L, Cheng J-X (2017). Quantification of lipid metabolism in living cells through the dynamics of lipid droplets measured by stimulated Raman scattering imaging. Anal. Chem..

[16] Dou W, Zhang D, Jung Y, Cheng J-X, Umulis DM (2012). Label-free imaging of lipid-droplet intracellular motion in early *Drosophila* embryos using femtosecond-stimulated Raman loss microscopy. Biophys. J..

[17] Marks DL, Vinegoni C, Bredfeldt JS, Boppart SA (2004). Interferometric differentiation between resonant coherent anti-Stokes Raman scattering and nonresonant four-wave-mixing processes. Appl. Phys. Lett..

[18] Hellerer T (2007). Monitoring of lipid storage in *Caenorhabditis elegans* using coherent anti-Stokes Raman scattering (CARS) microscopy. Proc. Natl. Acad. Sci. U.S.A..

[19] Di Napoli C (2016). Quantitative spatiotemporal chemical profiling of individual lipid droplets by hyperspectral CARS microscopy in living human adipose-derived stem cells. Anal. Chem..

[20] Wang P (2014). Imaging lipid metabolism in live *Caenorhabditis elegans* using fingerprint vibrations. Angew. Chem. Int. Ed.

[21] Yu Y, Ramachandran PV, Wang MC (2014). Shedding new light on lipid functions with CARS and SRS microscopy. Biochim. Biophys. Acta.

[22] Yue S (2014). Cholesteryl ester accumulation induced by PTEN loss and PI3K/AKT activation underlies human prostate cancer aggressiveness. Cell Metab..

[23] Li J (2017). Lipid desaturation is a metabolic marker and therapeutic target of ovarian cancer stem cells. Cell Stem Cell.

[24] Benalcazar WA, Boppart SA (2011). Nonlinear interferometric vibrational imaging for fast label-free visualization of molecular domains in skin. Anal. Bioanal. Chem..

[25] Rocha-Mendoza I, Langbein W, Borri P (2008). Coherent anti-Stokes Raman microspectroscopy using spectral focusing with glass dispersion. Appl. Phys. Lett..

[26] Langbein W, Rocha-Mendoza I, Borri P (2009). Coherent anti-Stokes Raman micro-spectroscopy using spectral focusing: Theory and experiment. J. Raman Spectrosc..

[27] Fu D, Holtom G, Freudiger C, Zhang X, Xie XS (2013). Hyperspectral imaging with stimulated Raman scattering by chirped femtosecond lasers. J. Phys. Chem. B.

[28] Coupé P, Munz M, Manjón JV, Ruthazer ES, Collins DL (2012). A CANDLE for a deeper *in vivo* insight. Med. Image Anal..

[29] Tu H (2016). Stain-free histopathology by programmable supercontinuum pulses. Nat. Photonics.

[30] You S (2018). Intravital imaging by simultaneous label-free autofluorescence-multiharmonic microscopy. Nat. Commun..

[31] Zhang C, Boppart SA (2020). Dynamic signatures of lipid droplets as new markers to quantify cellular metabolic changes. Anal. Chem..

[32] Youle RJ, Van Der Bliek AM (2012). Mitochondrial fission, fusion, and stress. Science.

[33] Eisner V, Picard M, Hajnóczky G (2018). Mitochondrial dynamics in adaptive and maladaptive cellular stress responses. Nat. Cell Biol..

[34] Kubli DA, Gustafsson ÅB (2012). Mitochondria and mitophagy: the yin and yang of cell death control. Circ. Res..

[35] Youle RJ, Narendra DP (2011). Mechanisms of mitophagy. Nat. Rev. Mol. Cell Biol..

[36] Kim I, Rodriguez-Enriquez S, Lemasters JJ (2007). Selective degradation of mitochondria by mitophagy. Arch. Biochem. Biophys.

[37] Pickles S, Vigié P, Youle RJ (2018). Mitophagy and quality control mechanisms in mitochondrial maintenance. Curr. Biol..

[38] Lee MP, Gear AR (1974). The effect of temperature on mitochondrial membrane-linked reactions. J. Biol. Chem..

[39] Pamenter ME, Lau GY, Richards JG (2018). Effects of cold on murine brain mitochondrial function. PLoS ONE.

[40] Watmough N (1990). Impaired mitochondrial beta-oxidation in a patient with an abnormality of the respiratory chain. Studies in skeletal muscle mitochondria. J. Clin. Invest..

[41] Wang Y (2019). Mitochondrial fatty acid oxidation and the electron transport chain comprise a multifunctional mitochondrial protein complex. J. Biol. Chem..

[42] Zoladz JA (2017). Effect of temperature on fatty acid metabolism in skeletal muscle mitochondria of untrained and endurance-trained rats. PLoS ONE.

[43] Slipchenko MN, Le TT, Chen H, Cheng J-X (2009). High-speed vibrational imaging and spectral analysis of lipid bodies by compound Raman microscopy. J. Phys. Chem. B.

