# Supplementary material for: Tracking the formation and degradation of fatty-acid-accumulated mitochondria using label-free chemical imaging
Source: Sci Rep. 2021 Mar 23;11:6671. doi: 10.1038/s41598-021-85795-1 (PMC7988176; doi:10.1038/s41598-021-85795-1)
Supplement: Supplementary file 1 — Supplementary Information 1. [file 41598_2021_85795_MOESM1_ESM.docx]

## Supplementary information

## Tracking the formation and degradation of fatty-acid-accumulated mitochondria using label-free chemical imaging

Chi Zhang^1#^ and Stephen A. Boppart^1,2,3,4,5*^

^1^Beckman Institute for Advanced Science and Technology

^2^Department of Bioengineering

^3^Department of Electrical and Computer Engineering

^4^Cancer Center at Illinois

^5^Carle Illinois College of Medicine

University of Illinois at Urbana-Champaign

Corresponding author

* [boppart@illinois.edu](mailto:boppart@illinois.edu)

**Supplementary figures**


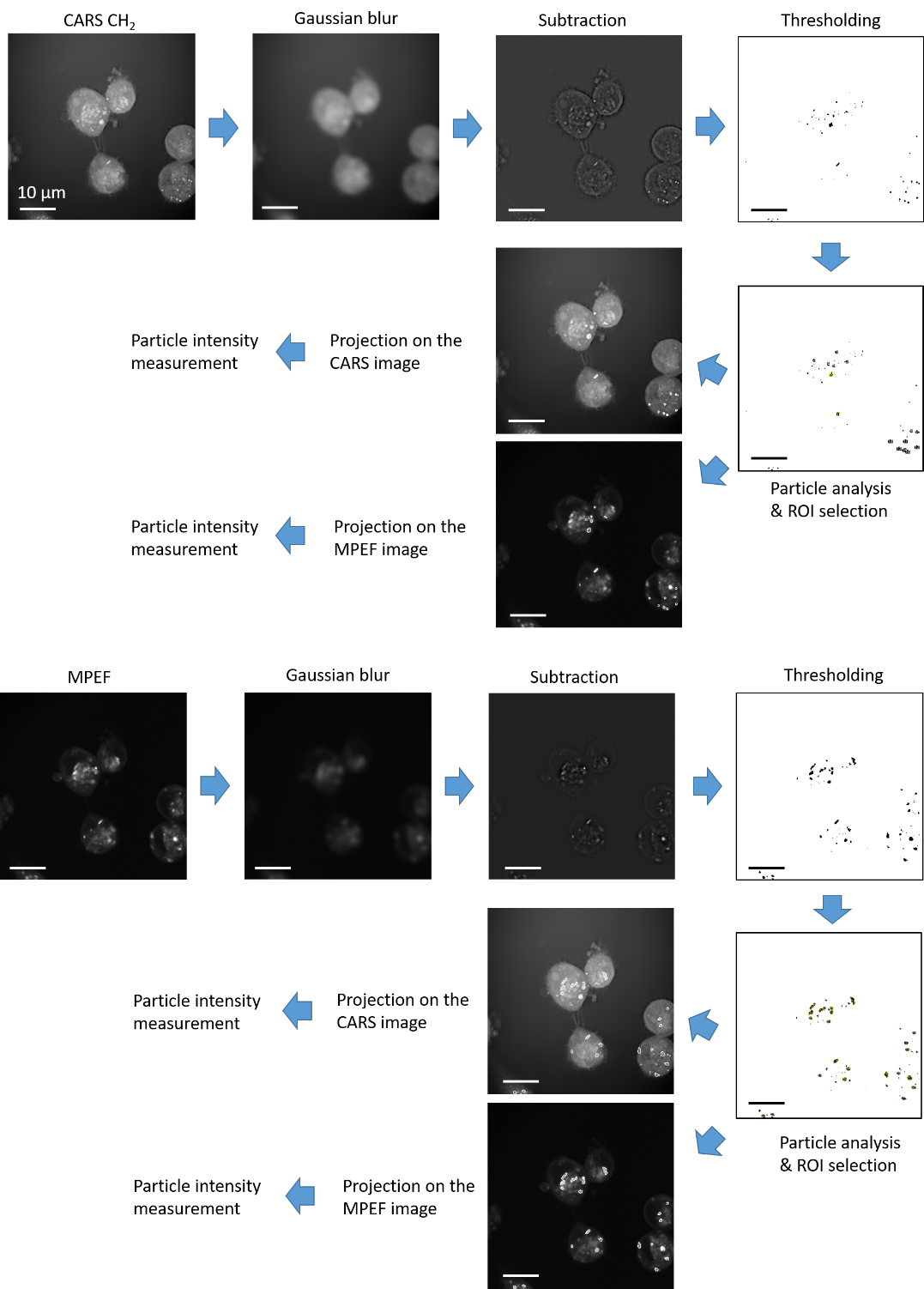


Figure S1. Method for quantitative analysis of particles using CARS and MPEF images. For CARS, the image was first processed with Gaussian blur (r=2 for 5 times). Then the original CARS image was subtracted by the blurred image to generate a subtraction image. Intensity thresholding was performed followed by particle analysis by ImageJ and region-of-interest (ROI) determination. Next, the ROIs were projected onto the original CARS and MPEF images for particle intensity measurement. The MPEF image analysis was performed with similar procedures.


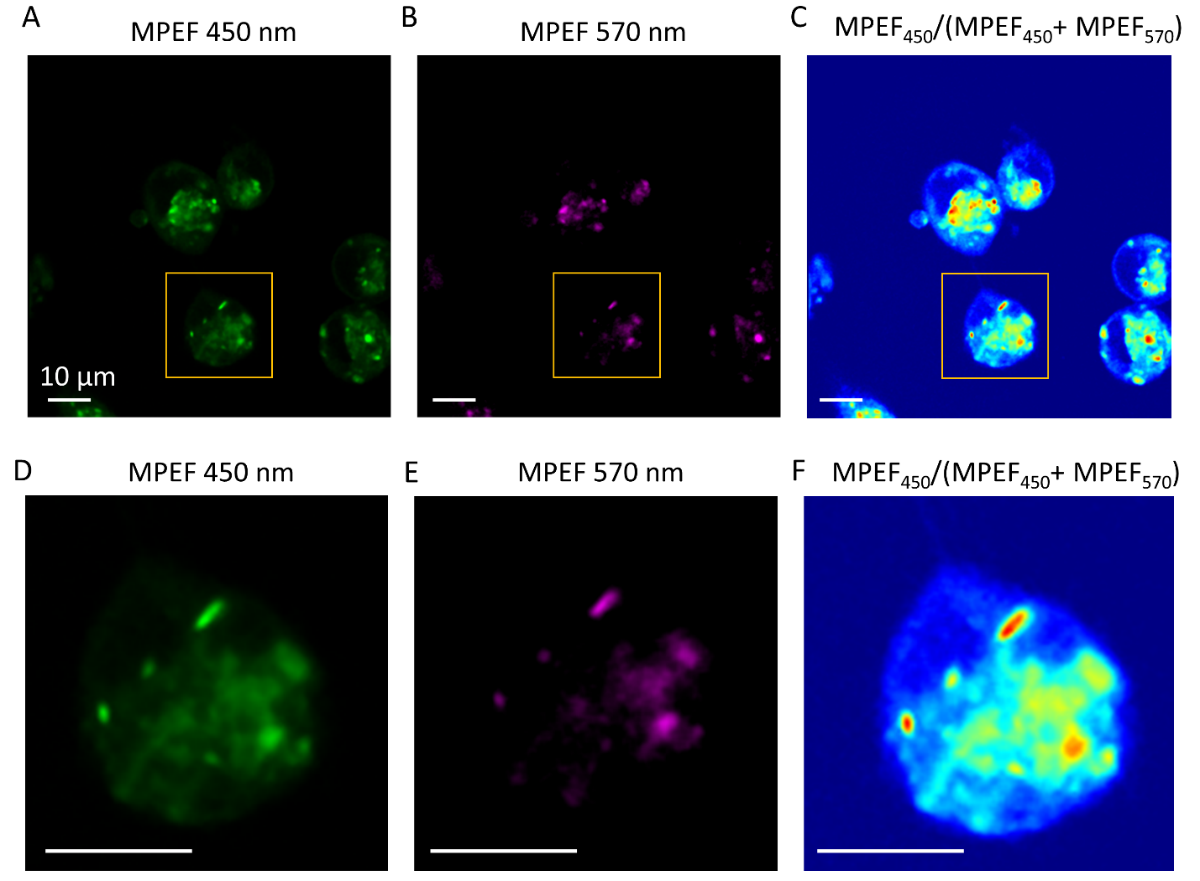


Figure S2. (A-C) MPEF images of MIAPaCa-2 cells at 450 nm (from NADH) and 570 nm (from FAD), and the intensity ratio of MPEF_450nm_/(MPEF_450nm_+MPEF_570nm_), respectively. The ratio has an arbitrary unit. (D-F) Magnified selected areas from the yellow boxed regions in panels (A-C), respectively. Panels C and F are displayed using the Jet colormap.

**Supplementary videos**


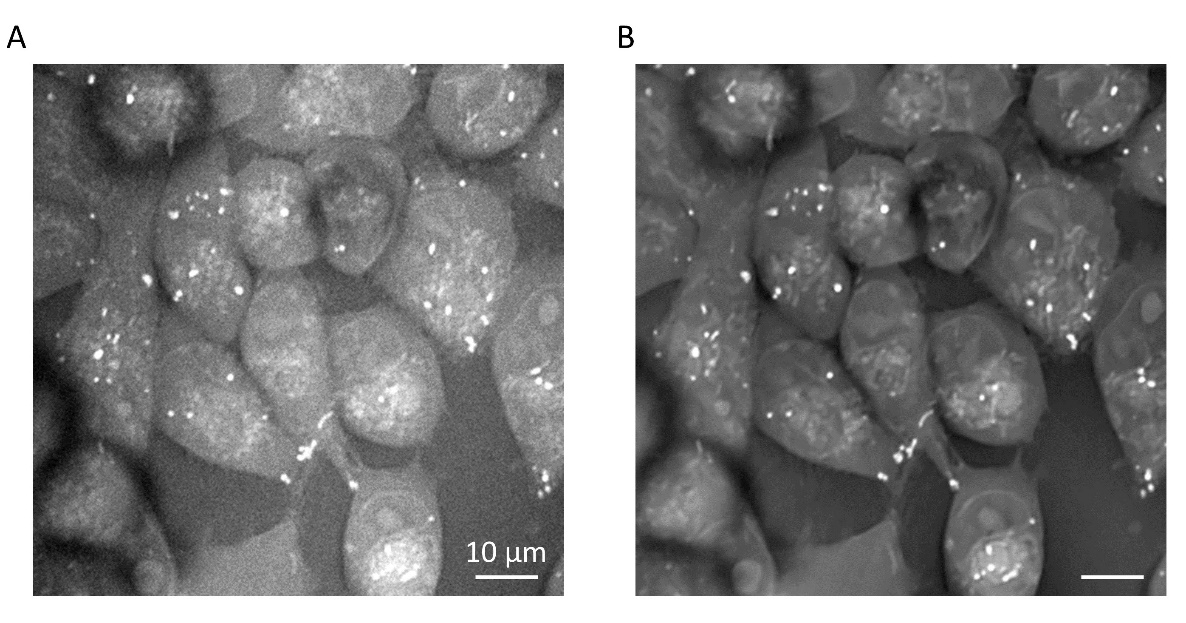


Video S1. (A) and (B) are the original and denoised CARS videos of MIAPaCa-2 cells at CH_2_ stretching vibrations, respectively.


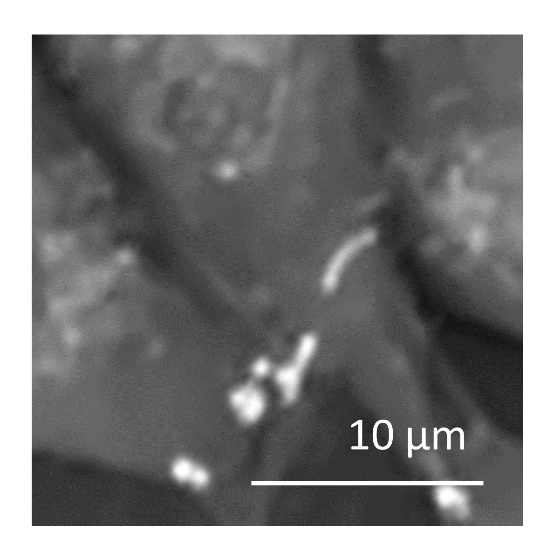


Video S2. A time-lapse video of the FA-accumulated mitochondria relocation in living cells.


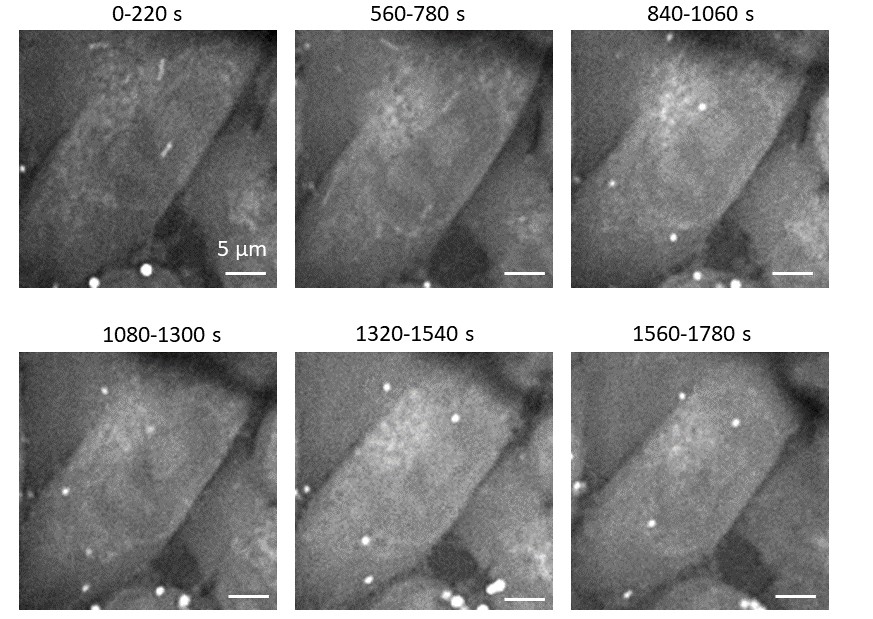


Video S3. Time-lapse videos of the FA-accumulated mitochondria degradation in a live MIAPaCa-2 cell. From 0 to 220 s, the cells were in the hypothermia environment. At ~380 s, the rewarming started.
